# Tanshinone IIA alleviates chondrocyte apoptosis and extracellular matrix degeneration by inhibiting ferroptosis

**DOI:** 10.1515/biol-2022-0666

**Published:** 2023-08-07

**Authors:** Jin Xu, Xiaocheng Zhi, Yunhui Zhang, Ren Ding

**Affiliations:** Department of Orthopaedics, Baoshan District Shanghai Integrated Traditional Chinese and Western Medicine Hospital, Baoshan District, Shanghai, 201999, China; Department of Orthopaedics, Baoshan District Shanghai Integrated Traditional Chinese and Western Medicine Hospital, No 181 You Yi Road, Baoshan District, Shanghai, 201999, China

**Keywords:** osteoarthritis, chondrocytes, oxidative stress, ferroptosis, Tanshinone IIA

## Abstract

Articular cartilage degeneration caused by chondrocyte damage is the primary pathological mechanism of osteoarthritis (OA). Oxidative stress is correlated with chondrocyte injury by potentiating ferroptosis, a newly identified form of cell death. Given the effects of Tanshinone IIA (Tan IIA) on alleviating oxidative stress, we further explored whether Tan IIA inhibited chondrocyte death and cartilage degeneration by decreasing ferroptosis. ATDC5 chondrocytes were treated with lipopolysaccharides (LPS) and Tan IIA, and cell viability was assessed using cell counting kit-8 (CCK-8) assays. Matrix metalloproteinase-13 (MMP13), a disintegrin and metalloproteinase with thrombospondin motif-5 (ADAMTS5), and type II collagen (Col II) levels were measured using quantitative real-time polymerase chain reaction (qRT‒PCR), western blotting, and immunofluorescence (IF) analysis. We demonstrated that Tan IIA treatment prominently increased ATDC5 cell viability and decreased cell apoptosis in the presence of LPS-induced stress. MMP13 and ADAMTS5 expression was increased, and Col II expression was decreased in ATDC5 cells after LPS stimulation, whereas these changes were reversed by Tan IIA. Mechanistically, Tan IIA inhibited LPS-induced ferroptosis in ATDC5 cells, as indicated by decreased levels of iron, reactive oxygen species, and malondialdehyde and increased GSH levels. Importantly, a ferroptosis agonist partially abrogated the effect of Tan IIA on alleviating chondrocyte damage and death. Taken together, these results suggest that Tan IIA ameliorates chondrocyte apoptosis and cartilage degeneration by inhibiting ferroptosis and may be a potential therapeutic agent for OA.

## Graphical abstract

**Figure:**
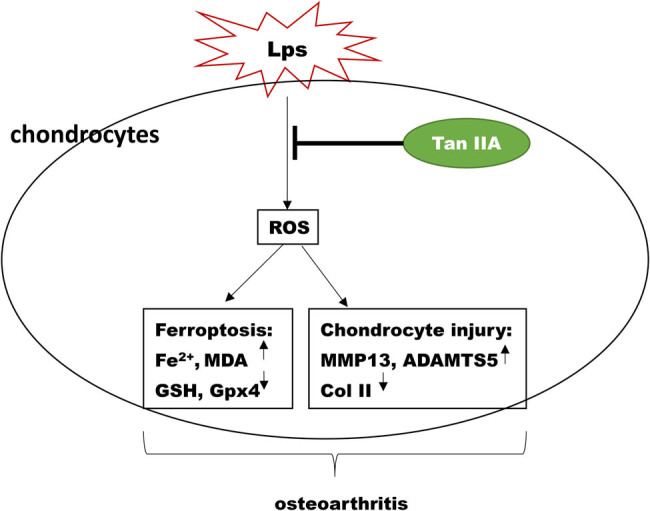

## 1 Introduction

Osteoarthritis (OA) is a frequent degenerative joint disorder worldwide that is characterized by intraarticular inflammation, cartilage degeneration, and architectural alterations in subchondral bone [1,2]. Ageing and obesity are the two major risk factors for OA in addition to genetic factors [3]. Chondrocytes, which originate from mesenchymal stem cells, are resident cells in articular cartilage that are responsible for extracellular matrix (ECM) generation [4,5,6]. Inflammatory factors, such as lipopolysaccharide (LPS) or IL-1β, accelerate chondrocyte apoptosis and subsequent cartilage degeneration by increasing the generation of proinflammatory factors and matrix metalloproteinases [7,8].

Medical intervention is necessary for OA patients because of the limited self-healing capacity of chondrocytes after injury [9]. Nonsteroidal anti-inflammatory agents and opioids are effective in alleviating inflammation and pain in OA patients, but their side effects limit their clinical application [10]. Several active components extracted from plants exert protective effects against chondrocyte injury because of their antioxidant and anti-inflammatory properties. Ellagic acid, a natural polyphenol in fruits or nuts, alleviates oxidative stress and chondrocyte injury by activating nuclear factor erythroid 2-related factor 2 (Nrf2) signalling [11]. Quercetin, a kaempferol isolated from lotus leaves, exerts a protective effect on joints and IL-1β-induced chondrocyte injury by regulating autophagy [12].

Tanshinone IIA (Tan IIA), a lipophilic component of Salvia miltiorrhiza, exerts protective effects against tissue inflammation and fibrosis [13]. Tan IIA mitigates LPS-induced brain injury by inhibiting oxidative stress and the inflammatory response [14]. Tan IIA ameliorates sepsis-induced lung damage by inhibiting NF-κB signalling activation in pulmonary epithelial cells [15]. Recent studies have also revealed the biological role of Tan IIA in regulating the inflammatory response and chondrocyte injury in OA. Tan IIA contributes to cartilage regeneration and exerts a protective effect against chondrocyte dedifferentiation [16]. Tan IIA treatment alleviates LPS-induced inflammation and chondrocyte apoptosis by inhibiting forkhead box O-3 (FOXO3) expression [17].

Ferroptosis is a recently identified form of cell death that is triggered by iron-dependent lipid peroxidation [18,19]. Lipid peroxidation caused by glutathione exhaustion or glutathione peroxidase 4 (GPX4) inactivation is a typical characteristic of cell ferroptosis [20]. Given the antioxidant effect of Tan IIA and that oxidative stress is associated with chondrocyte injury by potentiating ferroptosis [21], our aim was to investigate whether Tan IIA can alleviate chondrocyte injury by regulating ferroptosis.

## 2 Materials and methods

### 2.1 Cell culture and treatment

The mouse chondrocyte cell line ATDC5 was obtained from the Cell Bank of RIKEN BioResource Center (Tsukuba, Japan) and cultured in DMEM (Invitrogen, CA, USA) containing 10% FBS in a 5% CO_2_ incubator. Prior to functional experiments, 10 µg/mL recombinant insulin (Merck, MA, USA) was used to treat ATDC5 cells to induce chondrogenesis, as previously described [22]. ATDC5 cells were treated with LPS (5 µg/mL) and Tan IIA (0, 10, 20, 40, 100, or 200 µM) or erastin (10 µM) for 48 h, and then, cell viability, apoptosis, and cartilage degeneration were assessed.

### 2.2 CCK8 assay

ATDC5 cells were seeded in 96-well plates (2 × 10^3^ cells/well) and treated with LPS and Tan IIA (or erastin) for 48 h. Subsequently, 10 µL of CCK-8 was added to each well for 1 h at 37°C. The absorbance was measured at 450 nm with a microplate reader (Molecular Device, CA, USA).

### 2.3 Terminal deoxynucleotidyl transferase dUTP nick end labeling (TUNEL) assay

TUNEL staining was used to measure cell apoptosis. Briefly, after being treated with LPS and Tan IIA (or erastin) for 48 h, ATDC5 cells were fixed with 4% PFA (Solarbio, Shanghai, China) for 20 min and stained with TUNEL reagent (Solarbio) according to the manufacturer’s suggestions. The apoptotic cells were dyed with TUNEL and observed with a fluorescence microscope (Leica, Wetzlar, Germany).

### 2.4 qRT‒PCR

Total RNA was extracted from ATDC5 cells after treatment with LPS and Tan IIA (or erastin) using TRIzol reagent (Invitrogen), digested with RNA-free DNase I (TaKaRa, Tokyo, Japan), quantitated with a Bioanalyzer 2100 (Agilent, CA, USA), and used to synthesize cDNA with M-MLV and Oligo(dT)16 (TaKaRa). Subsequently, qRT‒PCR was performed in triplicate using a OneStep RT‒PCR kit (QIAGEN, Duesseldorf, Germany) on an Agilent Mx3005P qPCR system (Agilent) under the following conditions: 95°C for 10 min, 38 cycles of 95°C for 15 s and 59°C for 25 s. Gene expression was calculated with the 2^−ΔΔCT^ method [23] after normalization to β-actin. All primers are listed in Table S1.

### 2.5 Western blotting

Total proteins were extracted from ATDC5 cells after treatment with LPS and Tan IIA (or erastin) with RIPA buffer (Merck), quantified by BCA assays (Abcam, CA, USA), and separated by 12% SDS‒PAGE. After being separated, the proteins were transferred onto PVDF membranes (Merck). Subsequently, the membranes were sealed with 3% BSA, washed three times with TBST, and incubated with primary antibodies against Bax (ab32503, Abcam), Bcl-2 (ab182858, Abcam), MMP13 (ab39012, Abcam), ADAMTS5 (AB41037, Abcam), Col II (ab34712, Abcam), and β-actin (ab8227, Abcam) for 2 h at room temperature. Then, the membranes were washed five times with TBST, incubated with HRP-labelled secondary antibodies against rabbit or mice (Abcam), and visualized with a Novex® ECL reagent (Merck).

### 2.6 IF Assay

ATDC5 cells were grown on glass slides, fixed with 4% PFA, permeabilized with 0.1% Triton X-100, and sealed with 10% goat serum. After being washed three times with PBS, the cells were treated with primary antibodies against Col II (ab34712) overnight at 4°C and then incubated with Goat Anti-Rabbit IgG H&L (Alexa Fluor^®^ 488) (ab150077) for 1 h in the dark. Cell nuclei were dyed using DAPI (Merck). IF images were captured with a DMI4000B fluorescence microscope (Leica).

### 2.7 Reactive oxygen species (ROS) assay

ATDC5 Cells were cultured in 24-well plates (0.5 × 10^4^ cells/well) and treated with 5 µg/mL LPS and 40 µM Tan IIA. Forty-eight hours later, the cells were incubated with DCFH-DA (10 μM, Thermo Fisher Scientific) in the dark for 30 min [24]. Images were observed with a DMI4000B fluorescence microscope.

### 2.8 Iron concentration

ATDC5 Cells were treated with 5 µg/mL LPS and 40 µM Tan IIA for 48 h, and then, the concentration of ferrous iron (Fe^2+^) was measured using a commercial assay kit (ab83366, Abcam) in accordance with the manufacturer’s suggestions.

### 2.9 Malondialdehyde (MDA) and glutathione (GSH) assay

ATDC5 Cells were treated with 5 µg/mL LPS and 40 µM Tan IIA for 48 h, and then, MDA and GSH levels were measured with a lipid peroxidation assay kit (ab118970) and a glutathione assay kit (ab65322), respectively, according to the manufacturer’s suggestions and previous studies [25].

### 2.10 Statistics

All data are presented as the mean ± SD from at least three replicates, and statistical analyses were performed with SPSS 20.0 (IBM, NY, USA) using one-way ANOVA followed by the Scheffé test. A *p*-value < 0.05 was considered a statistically significant difference.

## 3 Results

### 3.1 Tan IIA-alleviated LPS-induced chondrocyte apoptosis

**Figure 1:**
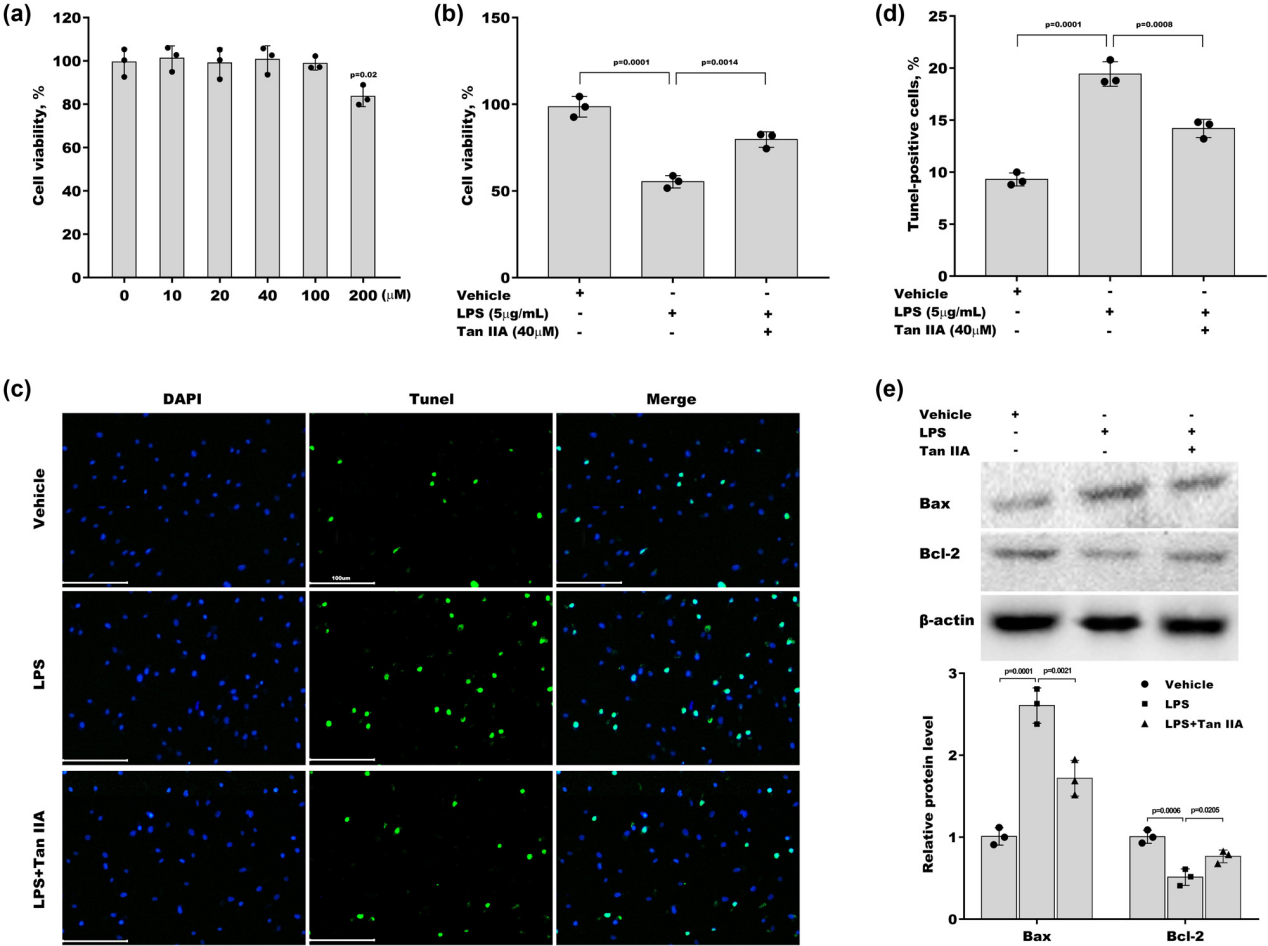
Tan IIA-alleviated LPS-induced chondrocyte apoptosis: (a) ATDC5 cells were treated with different doses of Tan IIA for 48 h, and cell viability was measured using CCK8 assays. (b) ATDC5 cells were treated with LPS in the presence or absence of Tan IIA for 48 h, and cell viability was measured using CCK8 assays. (c and d) ATDC5 cells were treated with LPS in the presence or absence of Tan IIA for 48 h, and cell apoptosis was measured using TUNEL staining. (e) ATDC5 cells were treated with LPS and Tan IIA for 48 h, and Bax and Bcl-2 protein levels were assessed using western blotting.

To investigate the role of Tan IIA in regulating chondrocyte viability, its cytotoxic effect was first measured. ATDC5 cells were treated with Tan IIA for 48 h, and cell viability was assessed using the CCK8 assay. Figure 1a shows that no cytotoxic effects were observed in response to Tan IIA at concentrations less than 100 μM. Subsequently, 40 μM Tan IIA was used to treat ATDC5 cells. Tan IIA treatment prominently rescued ATDC5 cell viability, which was repressed by LPS-induced stress (Figure 1b). The TUNEL assay revealed that Tan IIA prominently decreased LPS-induced ATDC5 cell apoptosis (Figure 1c and d). Moreover, there was an obvious increase in Bax protein levels and a decrease in Bcl-2 protein levels in ATDC5 cells in response to LPS-induced stress compared with the control, whereas these effects were blocked by Tan IIA (Figure 1e).

### 3.2 Tan IIA relieved LPS-induced chondrocyte injury

**Figure 2:**
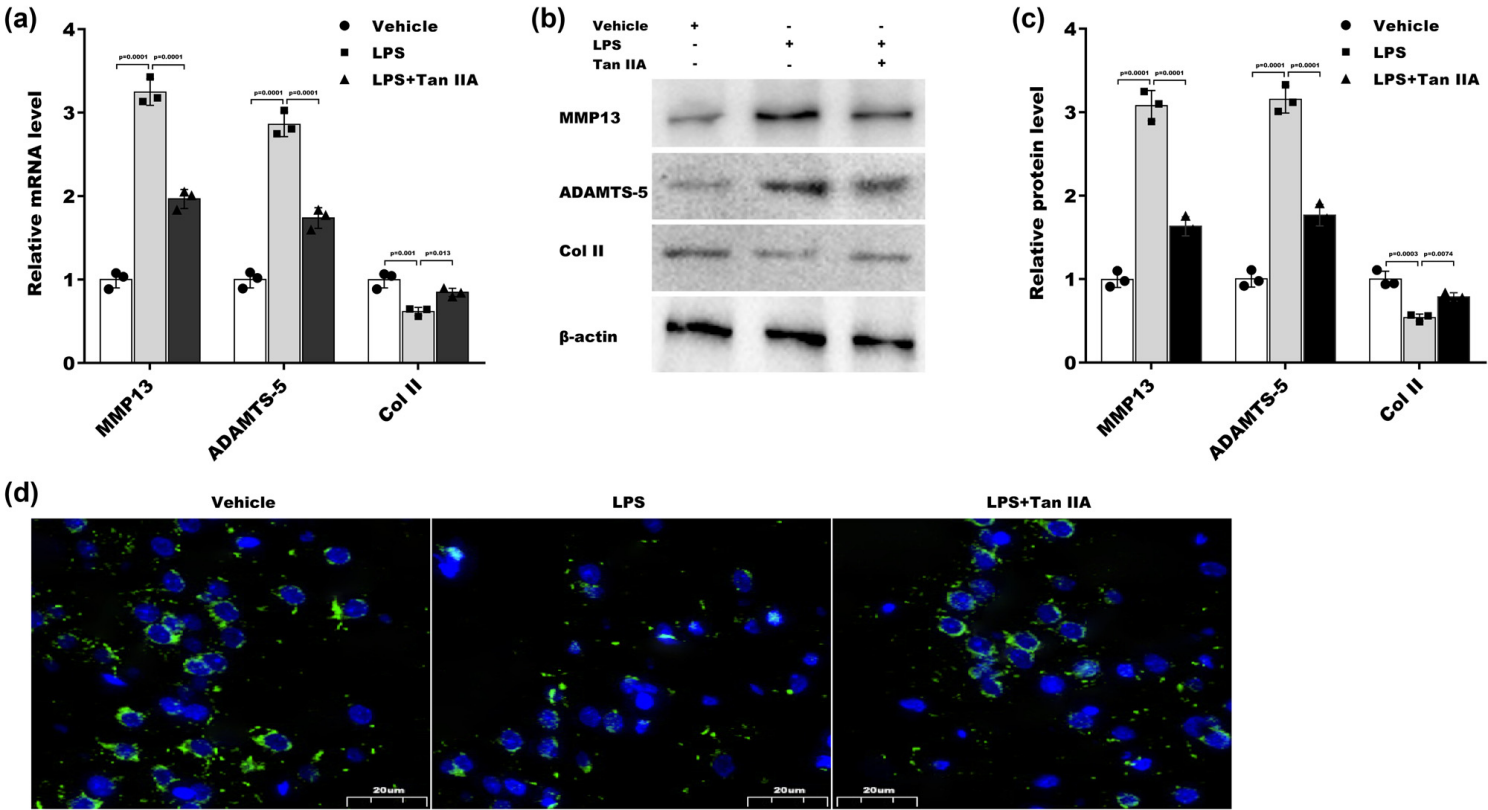
Tan IIA alleviated LPS-induced chondrocyte injury: (a) ATDC5 cells were treated with LPS and Tan IIA for 48 h, and MMP13, ADAMTS5, and Col II mRNA levels were assessed by qRT‒PCR. (b and c) ATDC5 cells were treated with LPS and Tan IIA for 48 h, and MMP13, ADAMTS5, and Col II protein levels were assessed by western blotting. (d) IF analysis of Col II in ATDC5 cells after treatment with LPS in the presence or absence of Tan IIA for 48 h.

To investigate the effect of Tan IIA on regulating LPS-induced chondrocyte injury, ATDC5 cells were treated with LPS in the presence or absence of Tan IIA, and then, the expression of cartilage-degrading enzymes (MMP13 and ADAMTS5) and Col II was assessed. The qRT‒PCR results revealed that MMP13 and ADAMTS5 mRNA levels were increased, and Col II mRNA levels were decreased in ATDC5 cells after treatment with LPS, whereas Tan IIA effectively suppressed these changes (Figure 2a). Furthermore, western blot analysis revealed similar effects of Tan IIA on regulating MMP13, ADAMTS5, and Col II protein expression in ATDC5 cells (Figure 2b and c). IF Staining also revealed that Col II levels were decreased in ATDC5 cells in response to LPS-induced stress, whereas Tan IIA inhibited these changes (Figure 2d).

### 3.3 Tan IIA-inhibited LPS-induced chondrocyte ferroptosis

**Figure 3:**
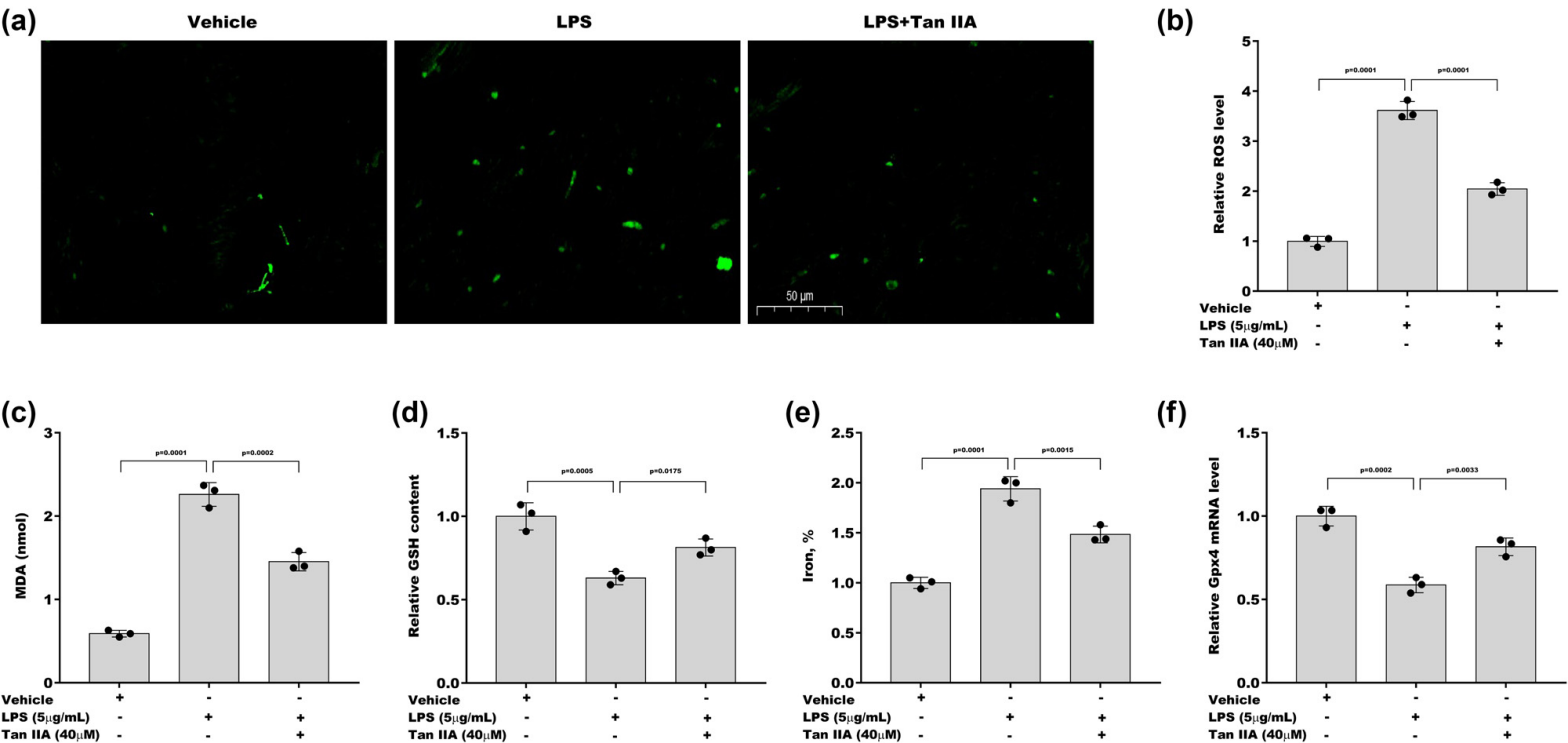
Tan IIA-inhibited LPS-induced chondrocyte ferroptosis. (a and b) Intracellular ROS levels were assessed in ATDC5 cells after treatment with LPS in the presence or absence of Tan IIA for 48 h. After treatment with LPS in the presence or absence of Tan IIA for 48 h, MDA levels (c), GSH levels (d), and iron concentration (e) were assessed in ATDC5 cells using commercial kits. (f) ATDC5 cells were treated with LPS and Tan IIA for 48 h, and Gpx4 mRNA levels were assessed using qRT‒PCR.

Given that oxidative stress is correlated with chondrocyte injury by potentiating ferroptosis and that Tan IIA can alleviate oxidative stress, the role of Tan IIA in regulating chondrocyte ferroptosis was next investigated. Figure 3a and b shows that Tan IIA prominently repressed LPS-induced ROS generation in ATDC5 cells. To determine the effect of Tan IIA on ferroptosis, ATDC5 cells were treated with LPS and Tan IIA, and then, ferroptosis markers (iron concentration, MDA level, and GSH content) were measured. Figure 3c shows that MDA levels were increased in ATDC5 cells after LPS treatment, whereas this effect was reversed by Tan IIA. In contrast, Tan IIA increased GSH levels in LPS-treated ATDC5 cells (Figure 3d). More importantly, the iron concentration was elevated in ATDC5 cells after LPS treatment, whereas this change was reversed by Tan IIA (Figure 3e). Tan IIA also restored Gpx4 expression in LPS-treated ATDC5 cells (Figure 3f). These results demonstrate that Tan IIA effectively suppresses LPS-induced ferroptosis.

### 3.4 Tan IIA-alleviated chondrocyte injury by inhibiting ferroptosis

**Figure 4:**
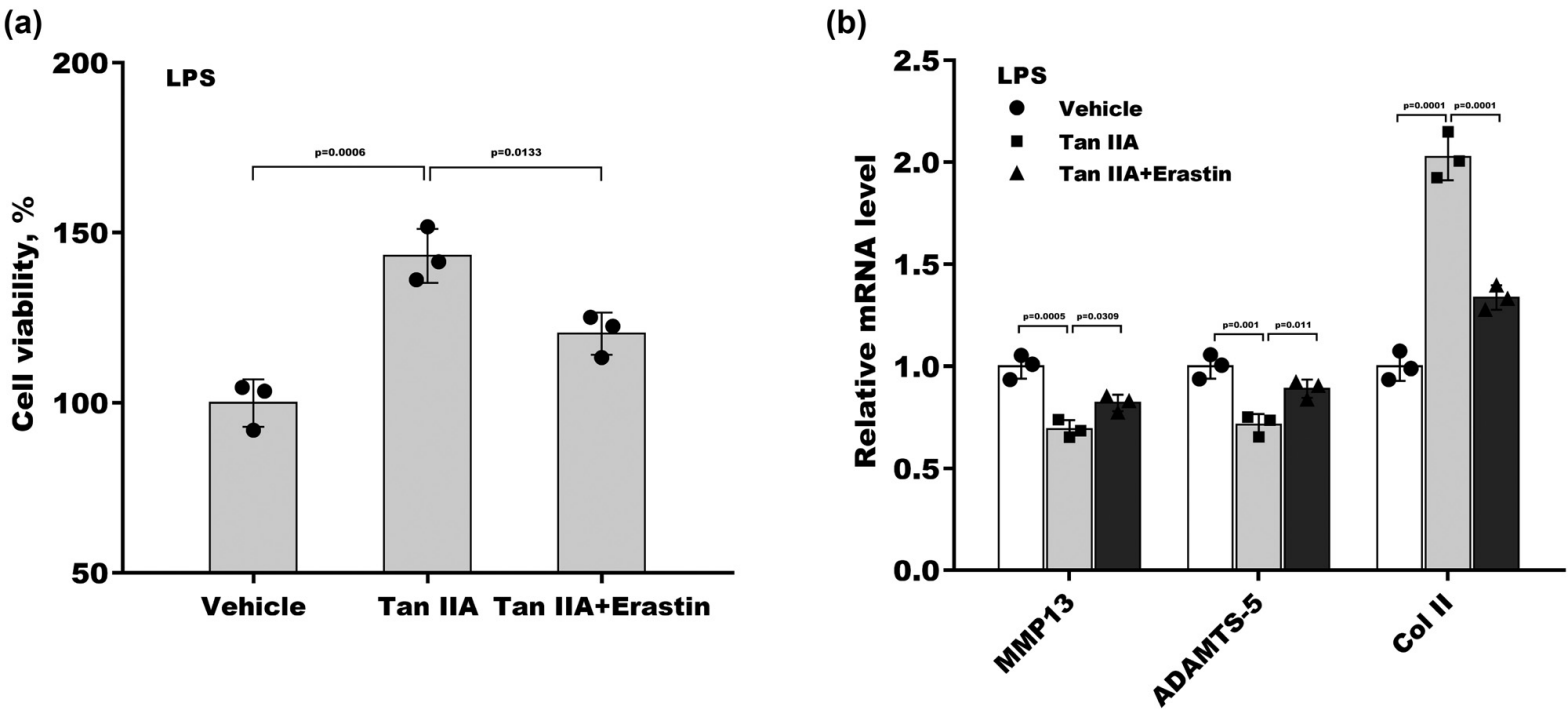
Tan IIA alleviates chondrocyte injury by inhibiting ferroptosis: (a) ATDC5 cells were treated with Tan IIA and erastin for 48 h under LPS-induced stress, and then, cell viability was measured using CCK8 assays. (b) ATDC5 cells were treated with Tan IIA and erastin for 48 h under LPS-induced stress, and then, the mRNA levels of MMP13, ADAMTS5, and Col II were assessed using qRT‒PCR.

Finally, the mediating role of ferroptosis in Tan IIA-regulating chondrocyte viability and injury was investigated. As shown in Figure 4a, although Tan IIA increased ATDC5 cell viability compared with the control in the presence of LPS, erastin (a ferroptosis agonist) treatment significantly abrogated this effect. Moreover, erastin suppressed the Tan IIA-induced decrease in MMP13 and ADAMTS5 levels and increase in Col II levels (Figure 4b), indicating that Tan IIA alleviates chondrocyte injury by repressing ferroptosis.

## 4 Discussion

Articular cartilage degeneration caused by chondrocyte injury is a major characteristic of OA, and oxidative stress plays a critical role in chondrocyte injury. Given the biological effect of Tan IIA on alleviating oxidative stress and inflammation, the protective effects of Tan IIA against inflammation-induced oxidative stress and chondrocyte injury were investigated. In this study, we demonstrated that i) Tan IIA mitigated LPS-induced chondrocyte apoptosis; ii) Tan IIA mitigated LPS-induced ECM degeneration; iii) Tan IIA mitigated LPS-induced oxidative stress and ferroptosis in chondrocytes, and iv) Tan IIA mitigated chondrocyte injury by inhibiting ferroptosis. These findings suggest that Tan IIA may be a promising therapeutic agent for treating OA.

ROS are chemically reactive molecules that are constantly produced in mitochondria by the reduction‒oxidation response or electronic excitation [26,27]. ROS generation is crucial for maintaining normal cellular function. Low ROS levels are necessary to sustain redox equilibrium, cell survival, and proliferation [28]. Cell ageing, the immune response, and inflammation are also managed by ROS [29,30,31]. ROS produced during inflammation and ageing contribute to the resorption of articular cartilage [32]. ROS concentrations are finely controlled by antioxidants under physiological conditions [31]. An imbalance in ROS production will result in oxidative stress and have dual effects on cell survival or death [33]. Elevated production of ROS destroys redox homeostasis and subsequently increases pro-death elements (e.g., Bax and Bid) and decreases pro-survival factors (e.g., Bcl-2) [33]. Low antioxidant levels are a hazard factor for OA owing to the overproduction of ROS. Therefore, synthetic and natural antioxidants may alleviate chondrocyte injury by scavenging ROS [32,34,35]. As an anti-inflammatory and antioxidant natural component in crustaceans, algae, and yeast, astaxanthin alleviates chondrocyte injury and slows OA progression by decreasing ROS generation [36]. Echinacoside, a natural phenylethanoid glycoside, increases chondrocyte viability and relieves OA by restraining oxidative stress [37].

Tan IIA is a phenanthrenequinone derivative in Salvia miltiorrhiza Bunge and exerts protective effects against tissue inflammation and fibrosis [13]. Tan IIA alleviates inflammation-induced brain injury by inhibiting oxidant stress and proinflammatory cytokines production [14]. Tan IIA inhibits inflammation-induced chondrocyte apoptosis by inhibiting FOXO3 expression [17]. In the present study, the effects of Tan IIA on regulating chondrocyte survival were further investigated. Tan IIA treatment decreased LPS-induced chondrocyte apoptosis. Tan IIA also relieved LPS-induced ECM degeneration by decreasing ROS generation.

In addition to apoptotic cell death, ROS can trigger ferroptosis in different types of cells [38,39,40], and ferroptosis is correlated with OA progression [40,41]. For example, Deferoxamine mitigates OA by decreasing chondrocyte ferroptosis [21]. Herein, we revealed the role of Tan IIA in inhibiting chondrocyte ferroptosis. In chondrocytes that were treated with LPS, ROS levels, MDA levels, and iron concentration were increased, while GSH levels and Gpx4 expression were decreased, indicating that LPS triggered chondrocyte ferroptosis. More importantly, these changes were reversed by Tan IIA. Furthermore, erastin abrogated the effect of Tan IIA on alleviating LPS-induced chondrocyte injury. The major limitations of this study were that i) the underlying mechanism by which Tan IIA regulates chondrocyte ferroptosis remains unclear, and ii) it is essential to investigate whether Tan IIA regulates other forms of cell death besides ferroptosis.

## 5 Conclusion

Tan IIA alleviates chondrocyte apoptosis and ECM degeneration by inhibiting ferroptosis.

## Supplementary Material

Supplementary Table
