# Supplementary material for: Tanshinone IIA alleviates chondrocyte apoptosis and extracellular matrix degeneration by inhibiting ferroptosis
Source: Open Life Sci. 2023 Aug 7;18(1):20220666. doi: 10.1515/biol-2022-0666 (PMC10426267; doi:10.1515/biol-2022-0666)
Supplement: Supplementary Table [file biol-2022-0666-sm.pdf]

Supplementary material

Table S1: Primer sequences

Target gene	Forward primer	Reverse primer
MMP13	GGAGCCCTGATGTTTCCCAT	ATCAAGGGATAGGGCTGGGT
ADAMTS-5	GCAAAACAGCCATACTGTGC	AACCATACAAGTGCCTTTTCTCT
collagen II	GCCAGGATGCCCGAAAATTAG	CGCACCCTTTTCTCCCTTGT
b-actin	ACTGTCGAGTCGCGTCCA	TCATCCATGGCGAACTGGTG
